# The proton and metal binding sites responsible for the pH-dependent green-red bioluminescence color tuning in firefly luciferases

**DOI:** 10.1038/s41598-018-33252-x

**Published:** 2018-12-04

**Authors:** Vadim R. Viviani, Gabriele V. M. Gabriel, Vanessa R. Bevilaqua, A. F. Simões, T. Hirano, P. S. Lopes-de-Oliveira

**Affiliations:** 1 Graduate Program of Biotechnology and Environmental Monitoring, Federal University of São Carlos (UFSCar), Rodovia João Leme dos Santos, km 110, Itinga, Sorocaba, SP Brazil; 2 Graduate Program of Evolutive Genetics and Molecular Biology, Federal University of São Carlos (UFSCar), São Carlos, SP Brazil; 3 Laboratório Nacional de Biociências (LNBio), Centro Nacional de Pesquisa em Energia e Materiais (CNPEM), Campinas, SP Brazil; 4 Tokyo Electrocommunication University, Tokyo, Japan

## Abstract

Firefly luciferases produce yellow-green light under physiological and alkaline conditions, however at acidic pH, higher temperatures or in the presence of heavy metals the color changes to red, a property called pH-sensitivity. Despite many decades of studies, the proton and metal binding sites responsible for pH-sensitivity remain enigmatic. Previously we suggested that the salt bridge E311/R337 keeps a closed conformation of the luciferin phenolate binding site. Here we further investigated the effect of this salt bridge and mutations of the neighbor residues H310 and E/N354, on metal and pH-sensitivity of firefly luciferases emitting distinct bioluminescence colors (*Cratomorphus distinctus*: 548 nm; *Macrolampis* sp2: 569 nm). The substitutions of H310 and E/N354 modulate metal sensitivity, whereas the carboxylate of E311 may work as the catalytic base essential for green bioluminescence and pH-sensitivity. Modeling studies showed that H310, E311 and E354 side-chains coordinate Zinc, constituting the metal binding site and the pH-sensor. Electrostatic potential and pKa calculations suggest that the external couple H310/E354 is affected by pH, whereas E311/R337 make a stabilized internal pair which retains excited oxyluciferin ejected proton near its phenolate group into a high energy state, promoting yellow-green bioluminescence. Protonation or metal binding weaken these electrostatic gates and their ability to retain the excited oxyluciferin released proton near its phenolate, promoting red light emission.

## Introduction

Luciferases are the enzymes which elicit the beautiful yellow-green flashes of fireflies during summer nights around the word. They catalyze an ATP-dependent oxidation of a benzothiazolic luciferin^1,2^. Whereas the normal bioluminescence color produced by firefly luciferases under physiological and alkaline conditions is usually yellow-green, at lower pH, in the presence of heavy metals such as Zn^2+^, Ni^2+^ and Hg^2+^ or at high temperatures, the color changes to bright orange-red, a property that has been called pH-sensitivity or pH-dependency^3,4^. Although many studies during the last decades have attempted to elucidate the mechanisms of bioluminescence color determination in beetle luciferases, the specific structural targets and mechanism of pH-sensitivity have remained elusive. Because firefly luciferases and their genes are widely used as bioanalytical reagents, reporter genes and biosensors^5,6^, and more recently are emerging as novel intracellular ratiometric biosensors for pH and heavy metals ions^7,8^, there is a lot of interest to better understand the origin of pH-sensitivity.

The color of bioluminescence depends on both the chemical structure of the emitter and on the surrounding luciferase active site microenvironment. The emitter, excited oxyluciferin, has potentially 6 forms including tautomers and anionic species^9–11^. Most recent experimental and theoretical studies indicate that the keto-phenolate form is the most likely emitter^11,12^, but the enol-phenolate and enolate-phenolate forms were also proposed as potential emitters^13–15^. Three general mechanisms have been quoted to explain bioluminescence color determination by the luciferase active site^7^: (I) non-specific solvent and polarizability effects^16,17^; (II) specific acid-base and electrostatic interactions of active site residues with excited oxyluciferin^9,18^ and (III) the conformation of the active site affecting the rigidity of the microenvironment and the rotation of thiazinic rings of excited oxyluciferin^19^. Among the specific interactions, the presence of bases near the thiazinic side of the luciferin binding site assisting the tautomerization between a keto and enol forms has been originally claimed to explain green to red bioluminescence color change in firefly luciferases^9,13^. More recently, interactions influencing the resonance forms of excited oxyluciferin^20^, specific acid-base and finally electrostatic effects around oxyluciferin 6′ phenol group are being considered to determine bioluminescence colors^18,21,22^.

More than 30 beetle luciferases have been already cloned, sequenced and investigated in the past 20 years^23–35^, most of them from fireflies. The three-dimensional structures have been solved for the North-American firefly luciferase *Photinus pyralis* (Ppy) in the absence of substrates^36^, in the presence of bromophorm^37^ and with the C-terminal trapped in a closed conformation^38^, for the Japanese *L. cruciata* firefly luciferase complexed with the luciferyl-adenylate analogue DLSA in a closed conformation and with oxyluciferin and AMP in an open conformation^39^, and finally, for *Lampyris turkanensis* luciferase^40^. The luciferin binding-site is well conserved among different beetle luciferases and consists mainly of the following residues and segments (*P. pyralis* numeration): R218, the motif 244HHGF247, the *loop* 314SGGAPLS320, and the β-hairpin motif 340YGLTETTS347^41–43^.

In firefly luciferases, several single-point mutations in the luciferin binding site and in other regions located far from the active site resulted in red mutants^41–46^, whereas a few of the respective mutations in the pH-insensitive click beetle and railroadworm luciferases affected the bioluminescence color^47–50^, indicating that the active sites of pH-sensitive firefly luciferases is somehow less rigid than that of pH-insensitive luciferases^50^. Mutation of the luciferin binding site residues, including histidines H244 and H245, which supposedly could assist oxyluciferin tautomerization in the thiazolyl part of the active site, resulted in red mutants in firefly luciferases^41–43^. However, H245 is invariant and H244 is conserved in beetle luciferases displaying distinct bioluminescence colors, therefore none of them could play the role of basic residues assisting tautomerization of excited oxyluciferin^13^. Furthermore, studies with the 5,5-dimethylluciferin-adenylate indicated that there is no need of C5 proton abstraction and tautomerization of oxyluciferin for pH-sensitive spectral changes^20^. Interactions of main-chain amide bonds with oxyluciferin phenolate^44^ and the active site conformation, were also proposed to be important for bioluminescence colors.

**Figure 1:**
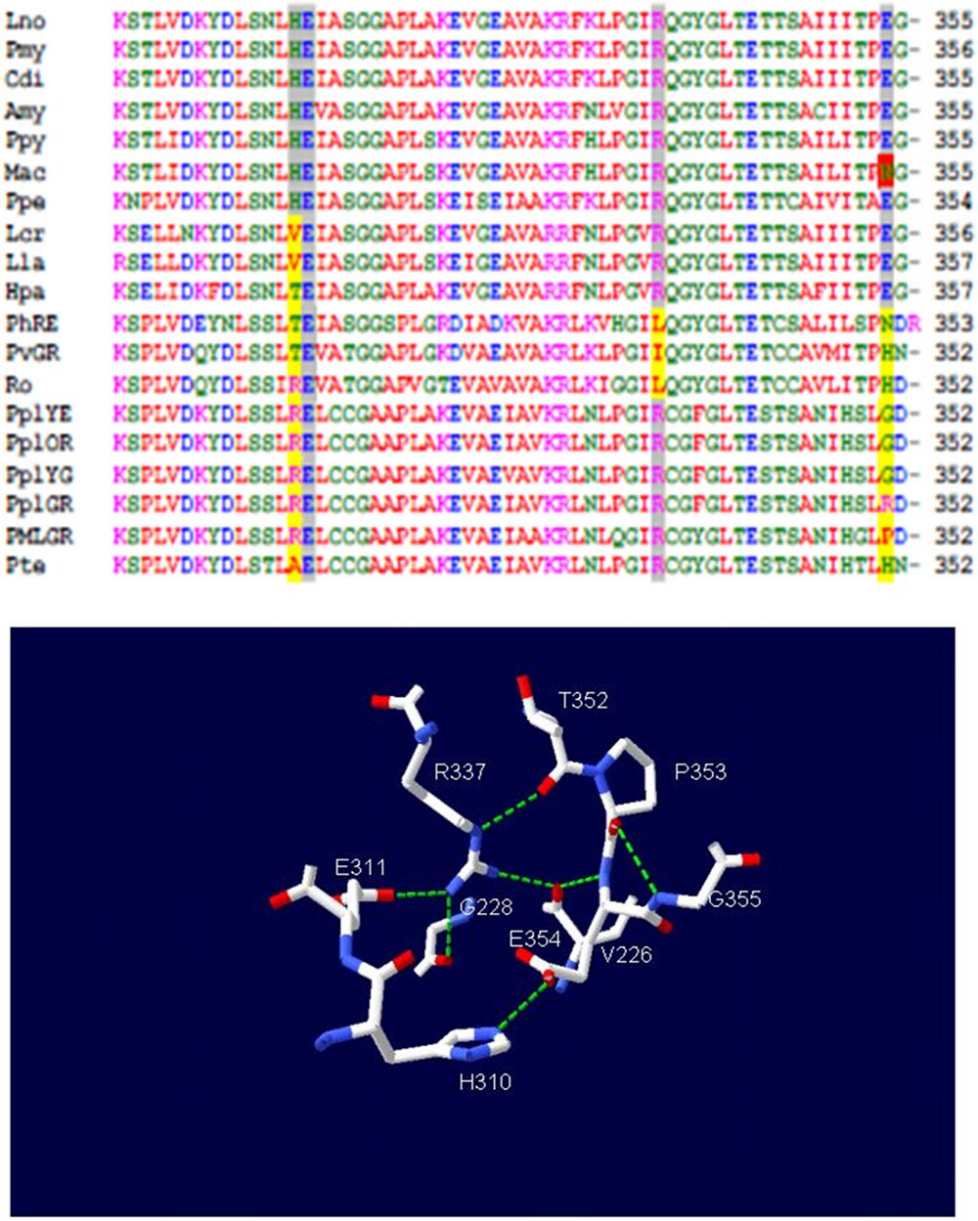
(Upper panel) Beetle luciferases primary structures multialignment, showing the residues H310, E311, R337 and E/N354 and interacting residues: (gray shadow) conserved residues of the pH-sensor in firefly luciferases; (yellow shadow) variable residues and (red shadow) unique substitutions affecting pH and metal sensitivity; (Lower panel) three-dimensional homology model of *Cratomorphus distinctus* firefly luciferase (83% identity with *P. pyralis* luciferase) showing E311/R337 and H310/E354 salt bridges and the network of polar interactions with the loop between residues 223–235 according to Viviani *et al*.^52^ which lead to the identification of the metal binding site.

Noteworthy, a group of mutations which affected spectra in both pH-sensitive and pH-insensitive luciferases was found to be clustered in the loops 223–235^51,52^ and 351–360^33,53^. The motif 227Y(F/V)GN(T)229 in the loop 223–235 was shown to be critical for bioluminescence colors and pH-sensitivity. In the loop 351–360, mutation of E354, which is conserved in the great majority of firefly luciferases, affected thermostability^54^ whereas the natural substitution E354N was shown to be responsible for the red-shifted, broader and more pH-sensitive bioluminescence spectrum in *Macrolampis sp2* firefly luciferase^33^. These loops, participate in a network of polar interactions with E311 and R337^20,53^ (Fig. 1), in the same bromophorm binding cavity described by Franks *et al*.^37^, leading to a first suggestion that this network could be the pH-sensor of firefly luciferases^52^. In this amphiphilic cavity, E311 and R337 are located internally close to oxyluciferin phenolate, and the residues H310 and E354 are located more externally. We recently provided evidences that the salt bridge between E311 and R337 is essential to stabilize a closed hydrophobic green emitting conformation in firefly luciferases^55^. However, the specific function of these residues and the identity of the specific targets responsible for pH- and metal-sensitivity remain unsolved.

**Figure 2:**
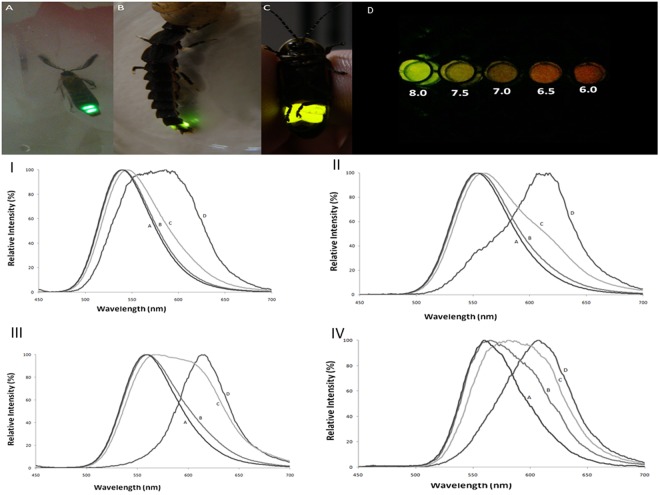
(Upper panel) Fireflies which emit different bioluminescence colors: (**A**) *Amydetes vivianii*firefly, (**B**) *Cratomorphus distinctus* larva and (**C**) *Macrolampis* sp_2_ firefly, and (**D**) pH effect on *in vitro* bioluminescence elicited by *Macrolampis* sp_2_ firefly luciferase. (Lower panel) Bioluminescence spectra of 4 firefly luciferases displaying distinct bioluminescence colors at different pHs: (I) *Amydetes vivianii*; (II) *Cratomorphus distinctus*; (III) *Photinus pyralis* and (IV) *Macrolampis* sp2; (a) 0.10 M Tris-HCl buffer pH 8.0; (b) 0.10 M Phosphate buffer pH 8.0; (c) phosphate buffer pH 7.0; (d) phosphate buffer pH 6.0.

Considering that H310, E311 and E354 display both prototropic and metal chelating side-chains, we decided to investigate whether they are involved in metal and pH-sensitivity. The mutation of H310 and N354 by other residues with metal chelating groups in *Macrolampis* firefly luciferase was recently shown to affect the metal-sensitivity in firefly luciferases^8^, indicating that this region is indeed critical for metal-sensitivity in firefly luciferases. Here we compared the effect of natural and artificial substitutions of these residues on metal- and pH-sensitivities of three Brazilian firefly luciferases displaying different bioluminescence colors (*Amydetes vivianii*: 539 nm; *Cratomorphus distinctus*: 548 nm and *Macrolampis* sp2: 569 nm; Fig. 2), modelled this region with Zinc ion, and calculated the associated electrostatic potential and pKas, providing convincing evidences that this region indeed constitutes the metal binding site and main pH-sensing moiety of firefly luciferases.

## Results

### Firefly luciferases bioluminescence spectra display different pH-sensitivities

When comparing the bioluminescence spectra of four firefly luciferases displaying different colors and pH-sensitivities (Fig. 2: *Amydetes vivianii*, 538 nm; *Cratomorphus distinctus*, 548 nm; *Photinus pyralis*, 558 nm, and *Macrolampis*, 564 nm), the bioluminescence spectrum of *Amydetes vivianii* luciferase was the most blue-shifted with the lowest amount of red light at pH 8.0, and is the less sensitive to pH, whereas that of *Macrolampis* sp2 luciferase has the broadest one with largest amount of red light even at pH 8, with *Photinus pyralis* and *Cratomorphus disctinctus* luciferases displaying intermediate values (Fig. 2). According to the solvent effect on the fluorescence spectra of luciferin and derivatives^16,17^, the results indicate that the active site of *Amydetes* luciferase is the most hydrophobic for both green and red emitters at pH 8 and 6. Recent quantum yield measurements by Ando *et al*.^56^ showed that the pH affects only the quantum yield of green emission, whereas the quantum yield of red emission is almost unaffected. Thus, we can assume that in other firefly luciferases too, only the amount of green emitter changes at different pHs. The results reinforce our previous proposal that in firefly luciferases the emission spectra are determined mainly by the ratio of green and red emitters^52^, although both green and red emitters may also experience slightly different environments in each luciferase, influencing the overall bioluminescence spectrum.

### Firefly luciferases display different metal sensitivities

Considering that the structural targets of sensitivity to pH, metal ions and temperature must be the same, and that firefly luciferases display different degrees of pH-sensitivity, we compared the effect of Zn^2+^, as a representative metal íon, on the bioluminescence spectra of the above firefly luciferases and their mutants. Similarly to the pH sensitivity, quantum yield measurements showed that the binding of these metal ions to *P. pyralis* luciferase induce decrease of the quantum yields of the green emission component, resulting in the remaining red emission spectra^57^.

**Figure 3:**
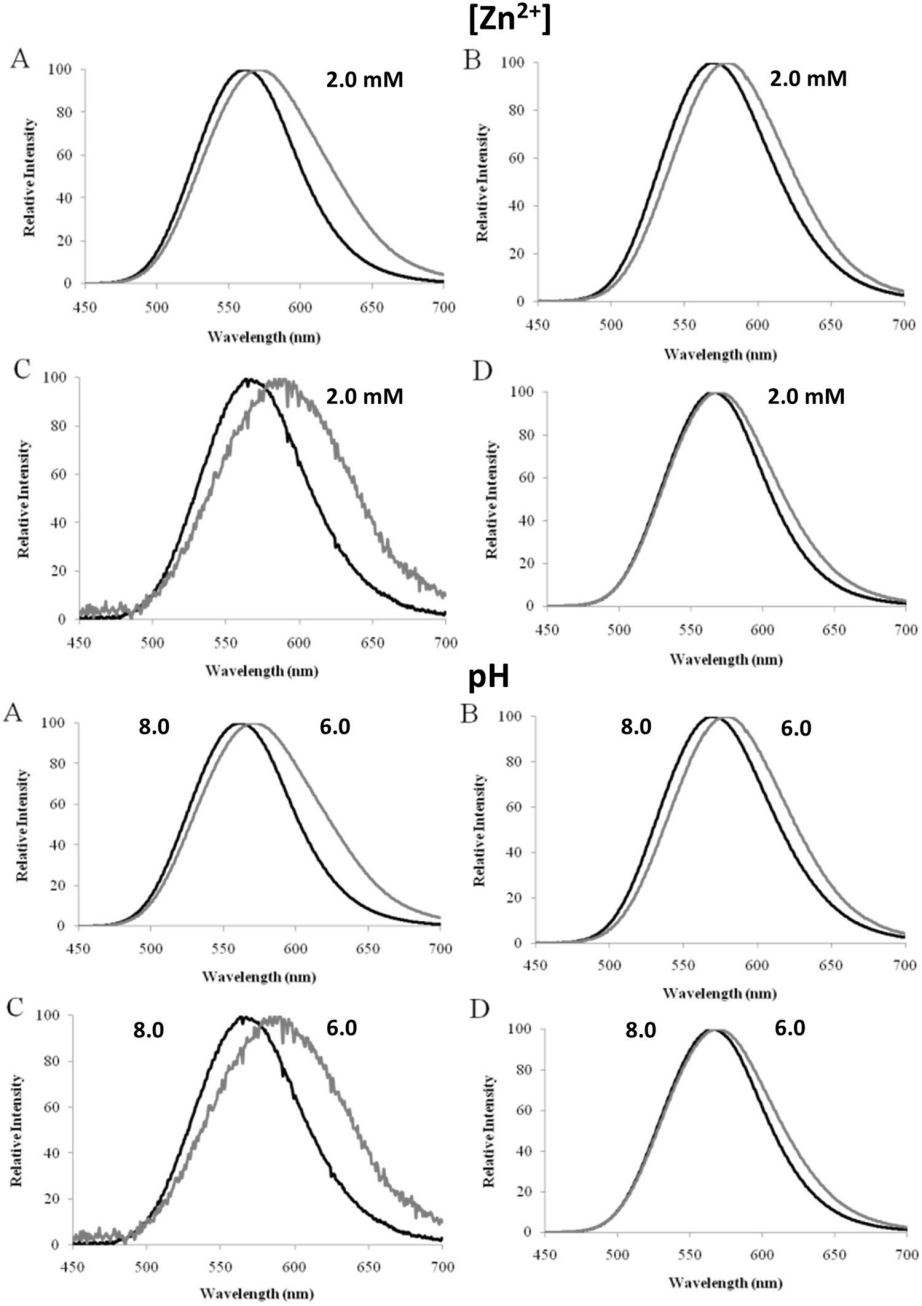
(Upper panel) Effect of ZnSO_4_ at the concentration of 2 mM (gray lines) in the bioluminescence spectra of distinct firefly luciferases and their mutants (black lines): (**A**) *Cratomorphus distinctus*; (**B**) *Macrolampis* sp2; (**C**) *Macrolampis* sp2 N354E; (**D**) *Cratomorphus distinctus* E354N; (Lower panel) Effect of pH on the bioluminescence spectra of *Macrolampis* firefly luciferase mutants: (**A**) wild-type; (**B**) Mac-H310C; (**C**) Mac-N354C and (**D**) Mac-N354H.

*C. distinctus* luciferase, similarly to *Photinus pyralis* luciferase, showed a larger red shift than *Macrolampis* sp2 luciferase in the presence of Zn^2+^ (Fig. 3). The luciferase of *Amydetes* firefly was much less to this metal (results not shown). Therefore, among these luciferases, that of *C. distinctus* was the most sensitive to these metal ions.

### Effect of E354 and H310 mutations on bioluminescence spectra and pH-sensitivity

Considering H310 and E354 are located close to E311 and R337, and that natural substitutions and mutations of E354 were shown to affect bioluminescence spectra and pH-sensitivity, we investigated the effect of mutations by residues which lack or replace the acid-base side chains of E354 and H310 in firefly luciferases pH-sensitivity. All mutants of H310 and E354 (H310A, H310C, N354C, N354H, N354E), independently of being substituted by acid-base or non-acid-base residues, still displayed pH-sensitivity (Fig. 3; Table 1). The wild-type *C. distintus* luciferase, was more sensitive to pH than *Macrolampis* luciferase, which naturally displays the substitution E354N. The most pH-sensitive mutants of *Macrolampis* firefly luciferase were N354C > N354E > H310C/N354C > N354H > WT.

**Table 1:** Summary of the effect of mutations of residues H310, E311, R337 and N/E354 (*P. pyralis* luciferase sequence numbers) in beetle luciferases bioluminescence spectra.

Luciferase	Relative Activity pH 8.0 (%)*	λ_max_ [Band.] (nm) **	λ_max_ [Band.] (nm) **	λ_max_ (nm) **	λ_max_ (nm) **
		pH 8.0	pH 6.0	1 mM Zn^+2^	Amino-luciferin
*Macrolampis sp2*	100	573 [99]	610 [79]	576	594
H310A		578 [99]	607 [77]	579	601
H310C	62	573	613	587	601
H310R		579 [105]	607 [69]	610	
E311A		621	—	—	597
E311Q	31	623 [67]	603 [66]	600	598
E311D	40	600 [63]	617 [60]	601	597
E311R	0	—	—	—	—
R337K	58	606 [63]	615 [53]	604	605
R337E	162	597 [93]	608 [95]	—	602
E311R/R337E	1.5	601 [80]	604	601	601
N354E	3.6	558 [83]	606 [77]		602
N354C	75	564	606	613	597
N354H	5.7	568	615	590	610
*Cratomorphus distinctus*					
WT	100	548 [71]	610 [95]	564	
E354N	100	556 [86]	603[62.5]	568	
*Amydetes vivianii*	100	546	590	555	584

*The relative activities were calculated for each mutant in relation to its wild-type luciferase (100%). **Estimated error in bioluminescence spectrum peak was ±2.5 nm.

Whereas the mutant H310A has little effect on the bioluminescence spectrum of *Macrolampis* sp2 luciferase at pH 8.0^33^, the mutation H310A in *Cratomorphus* luciferase, resulted in a considerable broadening and red-shifting of the spectrum (results not shown). The absence of effect of H310A in *Macrolampis* sp2 luciferase, which naturally has the substitution E354N, and the broadening effect of H310A in *Cratomorphus* luciferase, which has E354, supports the existence of a critical stabilizing interaction between H310 and E354 in *Cratomorphus* luciferase (and perhaps *P. pyralis* luciferase) which may stabilize a closed green-emitting conformation, differently from *Macrolampis* sp2 firefly luciferase which lacks the interaction and produces a broader and red-shifted emission. Furthermore, the mutation of H310R in *Macrolampis* sp2 luciferase, which inserts a permanent positive charge in this region, was previously shown to result in a very broad and red-shifted spectrum^20^, indicating that a positive charge at this position change the distribution of emission toward the red.

Altogether, these results indicate that although mutations the positions 310 and 354 influence spectral distribution, they are not determinants of pH-sensitivity.

### H310 and N354 are metal-sensitive sites

Considering that substitutions of E354 affect the bioluminescence spectrum and pH-sensitivity of firefly luciferases, we decided to investigate whether this position is also involved with metal sensitivity. The mutant Mac-N354E in *Macrolampis* firefly luciferase displayed a larger red-shift in presence of Zn^2+^, similar to that observed for the wild-type *C. distinctus* luciferase that naturally displays the substitution N354E (Fig. 3; Table 1). The reverse mutant, E354N in *C. distinctus* luciferase, had opposite effect, undergoing a smaller red shift, similar to that observed for the wild-type *Macrolampis* sp2 that displays N354 at this position. These results indicate that the position 354 is indeed important for metal sensitivity.

Since in *Cratomorphus distinctus* and *Photinus pyralis* luciferases E354 is at hydrogen bonding distance to H310, potentially making a salt bridge, and histidines have strong affinity to divalent metals, we also investigated whether the mutation of H310 affects metal sensitivity. The mutant H310A^8^, in which the metal chelating imidazole side-chain is removed, indeed was much less sensitive to metal ions, indicating that H310 is also important to bind metal ions such as Zn^2+^. These results prompted us to investigate the effect of other substitutions of H310 and N354 by residues with metal chelating side-chains such as His, Cys or Glu on the metal-sensitivity of *Macrolampis* firefly luciferase, resulting in a recently published paper in which we produced mutants with different sensitivities to Zn^2+^, Hg^2+^ Cd^2+^ and Ni^2+^ which could be used to ratiometrically estimate these metal concentrations^8^.

Altogether, the above results clearly indicate that H310 and especially E354 are important sites for metal-sensitivity. However, the fact that the mutation of these residues by residues with non-chelating side-chains does not completely abolish metal-sensitivity, indicate that these residues are not the only targets for metal binding, and that there must be at least another metal binding group in the neighborhood.

### Structure of the metal binding site

**Figure 4:**
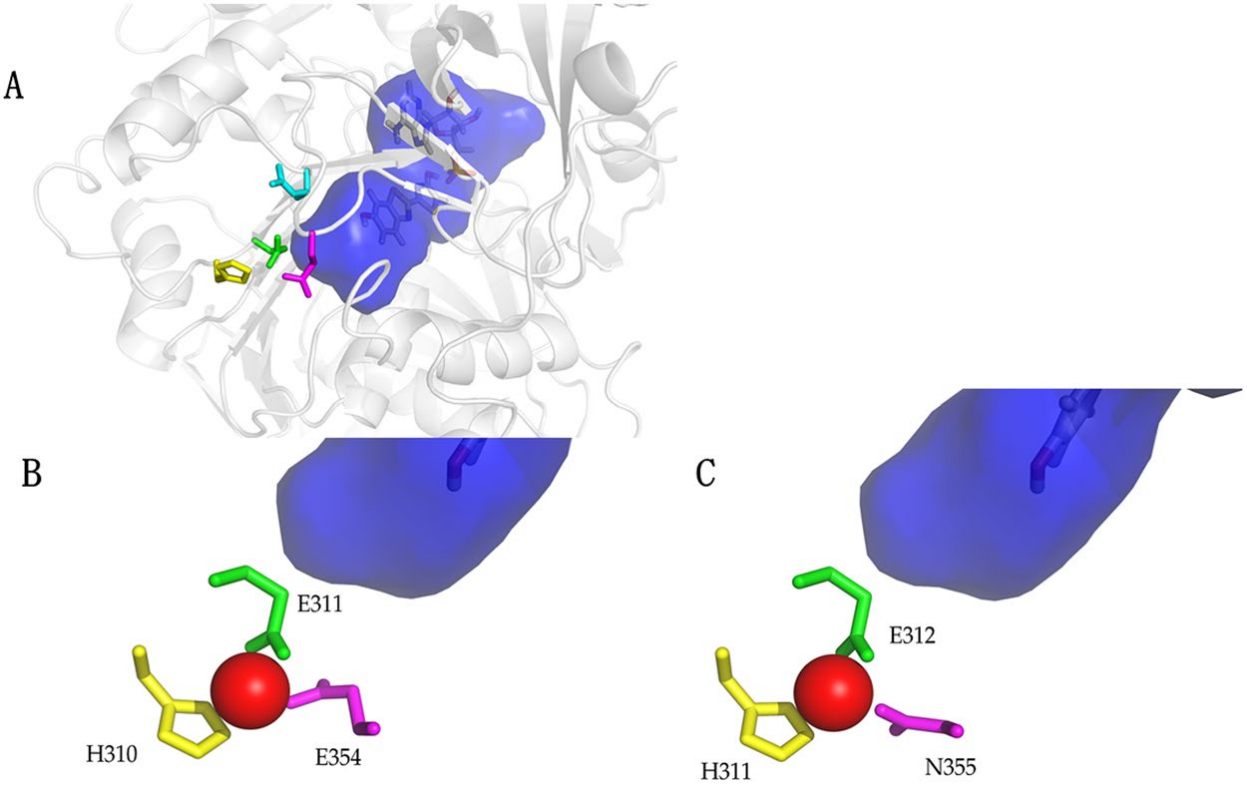
Structure of the metal binding site in firefly luciferases: (**A**) zoom showing *Cratmorphus distinctus* firefly luciferase luciferin binding site (dark blue) and metal binding site (colored side-chains); (**B**) zoom showing *Cratomorphus distinctus* firefly luciferase complexed with Zinc; (**C**) Zoom showing *Macrolampis* sp2 firefly luciferase complexed with Zinc. The dark blue area in the three figures represent the oxyluciferin phenolate binding pocket; yellow represent His310, green Glu 311 and pink Glu(Asn) 354.

Thus, in order to see whether the divalent heavy metal ions can bind to H310, E354 and surrounding region, modeling studies of beetle luciferases docked with Zn^2+^ were done (Fig. 4). Indeed, Zn^+2^ complexed with H310 N and carbonyl groups, with the side-chains of N354 in *Macrolampis* firefly luciferase or E354 in *Cratomorphus* firefly luciferase, and furthermore with E311 carboxylate, supporting the experimental results, showing that H310, E311 and E/N354 constitute the metal binding site.

### Role of the salt bridge between E311 and R337

Previously, we showed that the salt bridge between E311 and R337 is essential to stabilize the closed hydrophobic conformation in firefly luciferases, and that mutations which removed the charge or decreased the size of the side-chains, resulted in red light emission in firefly luciferases^54^. In order to investigate whether the salt bridge between E311 and R337 has just a conformational effect on bioluminescence color, or whether these residues display additional catalytic effects on bioluminescence color, we inverted the identity of these residues by constructing the double mutant E311R/R337E. Such double mutant is expected to keep the salt bridge, and therefore a closed conformation. If the color of bioluminescence was related exclusively to the maintenance of the closed conformation, we would expect that the double mutant would also produce yellow-green light at pH 8.0, such as the wild-type enzyme. However, the double mutant produced weak red bioluminescence (601 nm; Table 1). These results clearly indicate that green emission does not depend exclusively on a closed conformation kept by the salt bridge, but rather on additional specific interactions that such residues may play, especially E311.

### E311 is the main proton binding site

**Figure 5:**
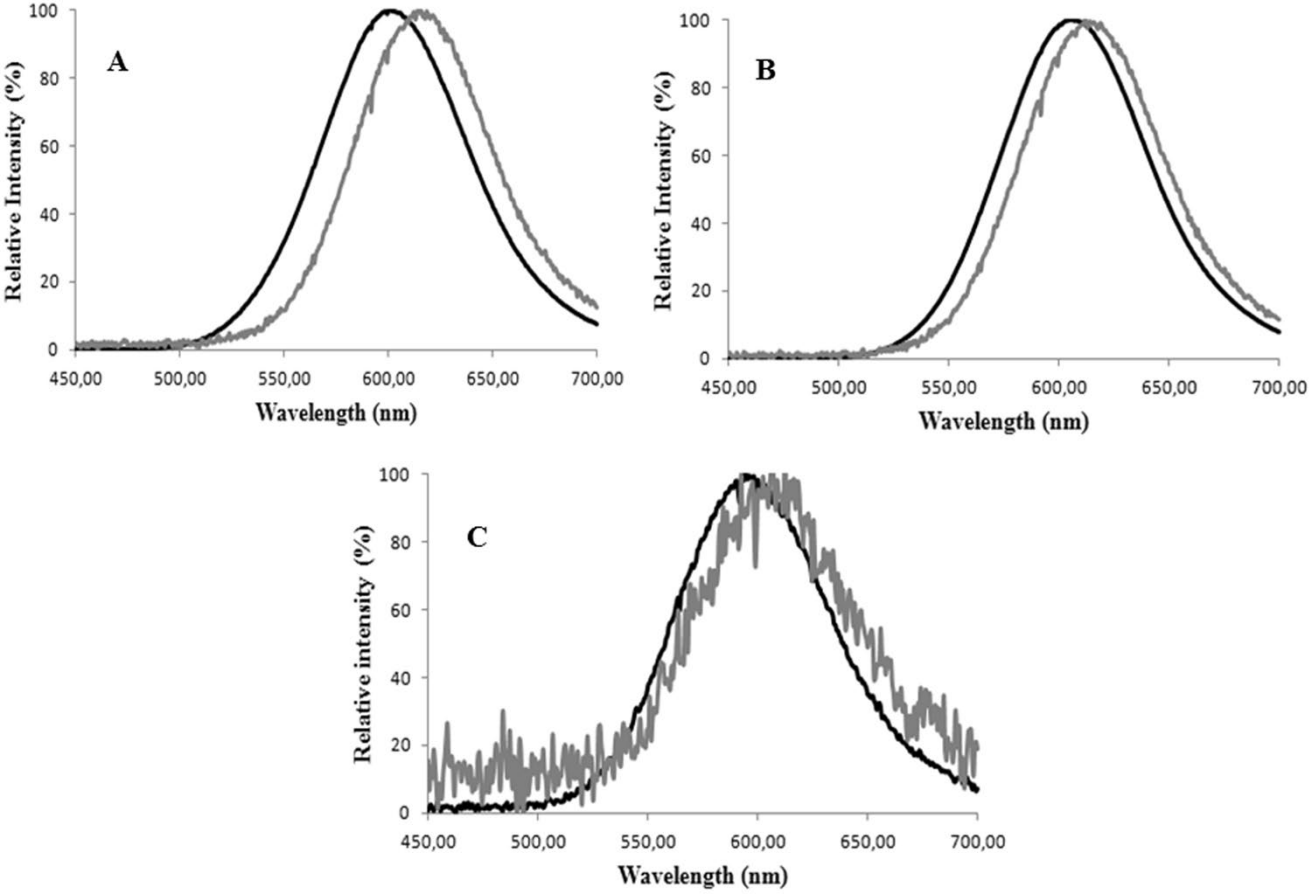
Effect of pH on the bioluminescence spectra of *Macrolampis* firefly luciferase mutants: (**A**) E311D; (**B**) R337K and (**C**) R337Q. (black) pH 8.0 and (gray) pH 6.0.

The above structural and site-directed mutagenesis results indicate that E311 display a key role in bioluminescence color. The mutants E311A and E311Q, in which the negative charge was removed, besides being the most red-shifted ones, are totally pH-insensitive and completely lost the bioluminescence activity at pH 6.0. The mutant E311R completely lost the activity, indicating that charge reversal is incompatible at this position. Only the mutants E311D and R337K, in which the side chains were shortened, but the charge preserved, still displayed a considerable degree of pH-sensitivity: at pH 6.0 E311D spectrum shifts about 17 nm to the red and R337K about 10 nm (Fig. 5). These results indicate that E311 negative charge is essential for green-yellow bioluminescence and also for pH-sensitivity in firefly luciferases. Furthermore, in contrast to R337 mutants, which independently of charge removal or reversal still produce weak red bioluminescence activity, the charge reversal of E311 (E311R), or removal (E311Q and E311A) under acidic pH, completely abolished the bioluminescent activity. Altogether, these results provide compelling evidences that E311 is the primary base responsible for green bioluminescence, and for proton and metal binding responsible for pH- and metal-sensitivityies, playing also a catalytic role in bioluminescence.

### Bioluminescence spectra of mutants with 6′Amino-luciferin

Previously we showed that the 6′-amino-luciferin, which has the 6′ hydroxil group substituted by an amino group, is a pH-insensitive luciferin analog which is useful to probe the oxyluciferin phenolate binding site microenvironment polarity^21^. Therefore, in order to check whether the mutations of H310 and N354 cause substantial polarity changes, we analyzed the bioluminescence spectra of this analog with mutants of these residues. However, the bioluminescence spectra peaks for all the mutants were very close to each other (Table 1), indicating that the microenvironment polarity is quite similar and is not considerably affected by these mutations.

### pKa analysis of the side-chains of pH-sensor

**Figure 6:**
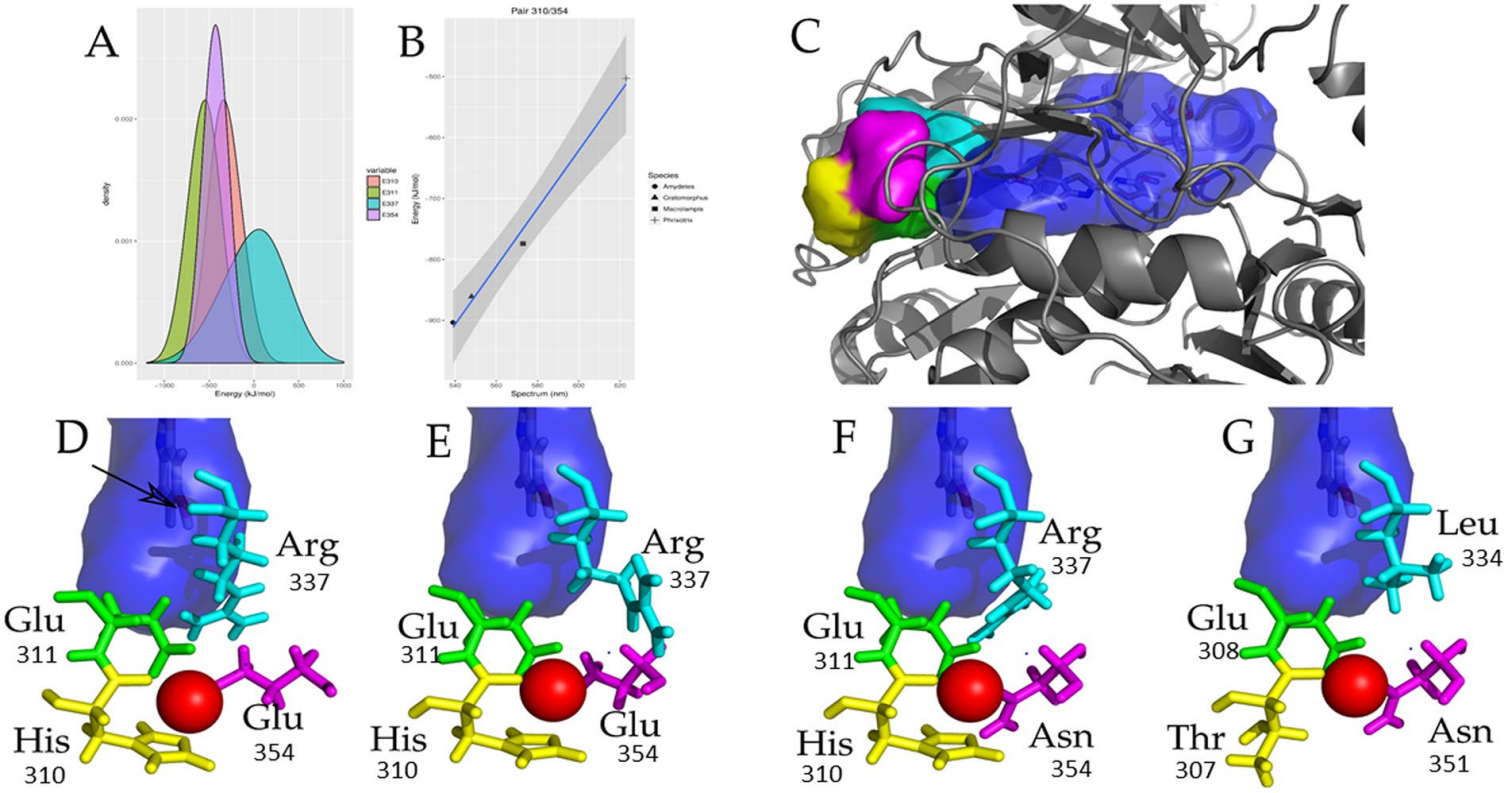
Electrostatic and van der Waals potential for the four pH-sensor residues microenvironment considering the solvent effects: (**A**) Energy distributions for all positions; (**B**) linear model showing the relationship between spectrum wavelength emission and electrostatic potential. Microenvironments of pH-sensor residues; (**C**) Structural relationships between pH-sensor microenvironment and active site (surface; blue) of four luciferases. Microenvironment for position 310 is represented in yellow, position 311 in green, position 337 in cyan and position 354 in magenta (numbering is based on *Cratomorphus distinctus* luciferase); (**D**–**G**) Representations for pH-sensor residues of *Amydetes vivianii*, *Cratomorphus distinctus*, *Macrolampis sp2* and *Phrixotrix vivianii* luciferases, respectively. Residues in each microenvironment are represented by sticks in the same color of microenvironment. Putative position for metal binding is indicated by red sphere. Arrow indicates position of phenolate involved in deprotonation. Volume of active site is 600 A^3^ ^66^.

In order to better understand the involvement of the above residues and their mutants in pH-sensitivity, we calculated the pKa of the identified residues in the wild-type luciferases and mutants, and correlated them with the electrostatic potential and bioluminescence spectra at different pHs and in the presence of metals (Fig. 6; Table 2 Supplemental Materials).

Among the wild-type residues, H310 was the one which displayed the largest shift of pKa value in relation to the reported value in water (~7.0), displaying a much more basic value (7.85 for *Macrolampis* luciferase and 8.95 for *Cratomorphus* luciferase). The pKa values for E311 and R337 were 4.53 and 13, respectively, close to the expected values in water.

As expected, the mutation of these residues affected the pKa of nearby side-chains. Only R337 pKa (13.0) was insensitive to mutations of nearby residues. Among the side-chains that had their pKa values affected by nearby mutations, again the imidazolium side-chain of H310 was the most sensitive site, changing several pH units upon mutations of E311 and R337, whereas the pKa of E311 carboxylate had its pKa only slightly changed upon the mutations of H310 and N354, but much less upon mutation of R337. The largest effects on the pKa of H310 were observed upon the mutations E311A and E311Q which removed the negative charge and decreased the value more than 3 pH units, below 5.4, whereas the mutation E311D, which preserved the negative charge, but with shorter side chain, also considerably lowered the pKa of H310. Altogether, these results are fully consistent with the need of the negative charge of E311 carboxylate to counterbalance the positive charge of R337 guanidinium group. Upon the removal of the negative charge of the E311 carboxylate by the mutations E311A or E311Q, the H310 imidazolium group becomes much more acidic, apparently to loose the extra positive charge in the neighborhood of the unshielded R337 guanidinium ion. Mutation of R337K, which preserved the positive charge but shortened the side chain, also had considerable lowering effect of H310 pKa. In agreement with the above results, charge reversal upon the mutation R337E resulted in a considerable increase of pKa of H310, which is consistent with the need of an additional neighbor positive charge in H310 to counterbalance the extra negative charge of E337. Altogether the results clearly indicate that all these side-chains are close enough to influence each other, especially E311 and R337 strongly influencing H310.

### Electrostatic potential calculation

To verify the effects of microenvironment in pH sensor residues, we calculated the electrostatic and van der Waals potential for each one of four putative residues considering the solvent effects. It can be observed that the positions 310 and 354 have similar contributions for pH-sensor site, whereas positions 311 and 337 display opposite potentials (Fig. 6-psloA). Furthermore, taking the total potential of four residues and the difference of these potentials at pH 6 and 8, unveil a clear relationship between increasing potential difference and a larger spectral shift, reinforcing the role of these residues on pH sensitivity.

To find the main potential components involved in the spectral change, the electrostatic potential for each candidate residue was combined in all possible pairs for the three wild-type pH-sensitive proteins of this study (*Amydetes vivianii*, *Cratomorphus distinctus*, *Macrolampis sp*_*2*_ firefly luciferases) and comparatively with the *Phrixotrix hirtus* railroadworm luciferase, which is an extreme model of pH-insensitive luciferase which naturally emits red light. At pH 8.0, of eleven evaluated regression models (Spectrum function as a function of energy), one model showed the best fitting: energy as the sum of electrostatic potential of the residue pair 310/354 (R2: 0.99) (Fig. 6_psloB). The green emitting luciferases of *Amydetes* and *Cratomorphus* display a stabilized electrostatic pair E311/R337, as indicated by favorable micro-environment (lowest potential value, about −900 kJ/mol). Here, the pair H310/E354 acts as strong acid-base pair, ultimately receiving the proton of phenolic hydroxyl group of oxyluciferin. In the more red-shifted *Macrolampis* luciferase, there is just one strong base (E312; equivalent to position 311 in *Photinus pyralis* luciferase) which makes an electrostatic pair with R338 (equivalent position to 337). Absence of the second base at position 355 (equivalent to position 354), increased the microenvironment potential, decreasing the strength to retain the proton ejected from excited oxyluciferin phenolate in the neighborhood. The red-emitting luciferase of *Phrixotrix* does not display the electrostatic pair (308/334 corresponding to 311/337) due the natural substitution of arginine by leucine (L334 corresponding to R337), and the entrance of this cavity (T307 and N351 corresponding to the couple H310/E354) do not have bases to assist residue E308 (equivalent to position 311) to trap oxyluciferin phenolate released proton. Not surprisingly, this luciferase displays the worst microenvironment potential (~−500 kJ/mol), with insensitivity to pH changes. Furthermore, although the mutation L334R slightly blue-shifts the spectrum of *Phrixotrix* luciferase, its microenvironment potential remained almost unaffected (−503 kJ/mol). Taken together, these results indicate that measurements of the microenvironment potential of pair 310/354 can provide a good estimate about pH sensitivity.

## Discussion

The structural identity of the pH- and metal-sensing moiety, as well as the underlying mechanisms of pH-sensitivity and bioluminescence colors in firefly luciferases, have remained elusive for decades.

Three mechanisms to explain pH-sensitivity have been proposed: (1) presence of basic residues near oxyluciferin C_5_^9^, promoting tautomerization, or phenolic hydroxyl group^12^ which could be protonated at lower pH; (2) conformational changes mediated by protonation of specific basic residues important to keep the active-site conformation, leading to entry of water molecules increasing the polarity of the oxyluciferin phenolate binding site^52^; (3) direct electrostatic interactions with oxyluciferin phenolate^53,57^. These hypotheses are not mutually exclusive.

The presence of bases, which has been originally suggested for the tautomerization hypothesis, are also required to accept the released proton from excited oxyluciferin phenol group. According to the hypothesis by Hirano *et al*.^12^, a base close to the phenolic hydroxyl group of oxyluciferin may serve to receive the proton, and the proximity of the conjugate acid to the excited oxyluciferin phenolate, may increase the degree of covalent bond character of the O···H bond between the phenolate group and the hydrogen of the conjugate acid. The compromise between the strength of the base and the polarity of the environment, may affect the degree of covalent character of the O···H bond and therefore modulating bioluminescence spectrum. Protonation of such bases at low pH may therefore affect bioluminescence spectra. However, any protic group, including water and main-chain amide groups, may serve as weak temporary bases to accept the proton ejected from the strongly acidic phenolic hydroxyl group of excited oxyluciferin (pKa ~ 1.0). The main-chain amide group of S/C/T314 (*Photinus pyralis* sequence number) indeed have already been shown to be important as putative assisting base near luciferin phenol group^44^.

pH-sensitivity was also explained on the basis of protonation of basic residues involved in keeping a closed active site conformation, promoting a conformational change to a more open and polar conformation, in the benzothiazolyl part of oxyluciferin binding site^53^. Nakatsu *et al*.^39^, showed that *L. cruciata* luciferase displays a close active site conformation with the critical luciferyl-adenylate intermediate analog, DLSA, and an open one with the products AMP and oxyluciferin. Later studies with 6′-aminoluciferin analogs supported the existence of two binding modes for oxyluciferin in firefly luciferases active site, an apolar (N) responsible for green light emission and another polar (P) responsible for red emission^21,22^. The active site in firefly luciferases may undergo conformational changes between these two extremes, and the light emitting step may take place somewhere between these two conformations. Whether such polar and apolar binding modes correspond to distinct conformations or sites remain to be elucidated.

Finally, electrostatic effects have been also claimed to play important roles in emission spectra modulation^53,57–59^. The presence of positive charges near oxyluciferin phenolate were suggested to blue shift the emission spectrum^53,57^. In this regard, the absence of an arginine at position 334 in the red emitting luciferase of *Phrixotrix* railroadworm luciferase was shown to be responsible for the very red-shifted spectrum of this enzyme, and inclusion of an arginine upon the mutation L334R (corresponding to R337 in *P. pyralis* luciferase) blue-shifted the emission^55^. In the pH-sensitive luciferases and their mutants, although the calculation of the overall microenvironment charge, considering the four residues H310, E311, R337 and E354, showed a trend for blue-shifted emission in electrostatically more negative environments and red-shifted emission for more positive environments (Table 2), the mutant R337E, in which there is a substantial increase of the overall negative charge character of the environment, also resulted in red light.

**Table 2:** Effect of mutations on the pKa values and potential of *Macrolampis* and *Cratomorphus* firefly luciferases (Supplemental Materials).

Luciferase/Mutant	pKa H310	pKa E311	pKa R337	pKa E/N354	Overall charge pH 8.0	Overall charge pH 6.0	λ_max_/[Band.] (nm)**
*Macrolampis sp*_*2*_ WT	7.85	4.53	13		0	1	573 [99]
H310A	—	3.54	13	—	0	0	578 [99]
H310C	8.7	3.54	13	—	0	0	573
H310R	13	3.3	13	—	1	1	573 [105]
E311A	4.93	—	13	—	1	2	621
E311Q	4.86	—	13	—	1	2	623 [67]
E311D	5.38	2.62	12	—	0	1	600 [63]
R337K	5.51	3.92	10.79	—	0	1	606 [63]
R337E	8.57	4.4	4.65	—	−2	−1	597 [93]
N354E	6.84	4.11	13	4.55	−1	0	558 [83]
N354C	7.13	3.89	13	8.7	0	1	564
N354H	7.11	3.68	13	7.12	0	2	568
*Cratomorphus*	8.9	5.0	13	4.65	−1	0	548 [71]
E354N	8.14	4.76	13		0	1	556 [86]

Here we show for the first time that the pH-sensing moiety and metal binding site responsible for pH-sensitivity involve the residues H310, E311, R337 and E(N)354. These four residues, with their acid-base side-chains are involved in two main electrostatic interactions that keep a closed and tight active site conformation favorable for green light emission: E311/R337 and H310/R354. The residues E311 and R337 make a more stable and internal salt bridge closer to oxyluciferin phenolate, whereas H310 and E354 are located in a more external and polar part of this cavity, making a second potential gate which is more susceptible to external pH and temperature changes.

**Figure 7:**
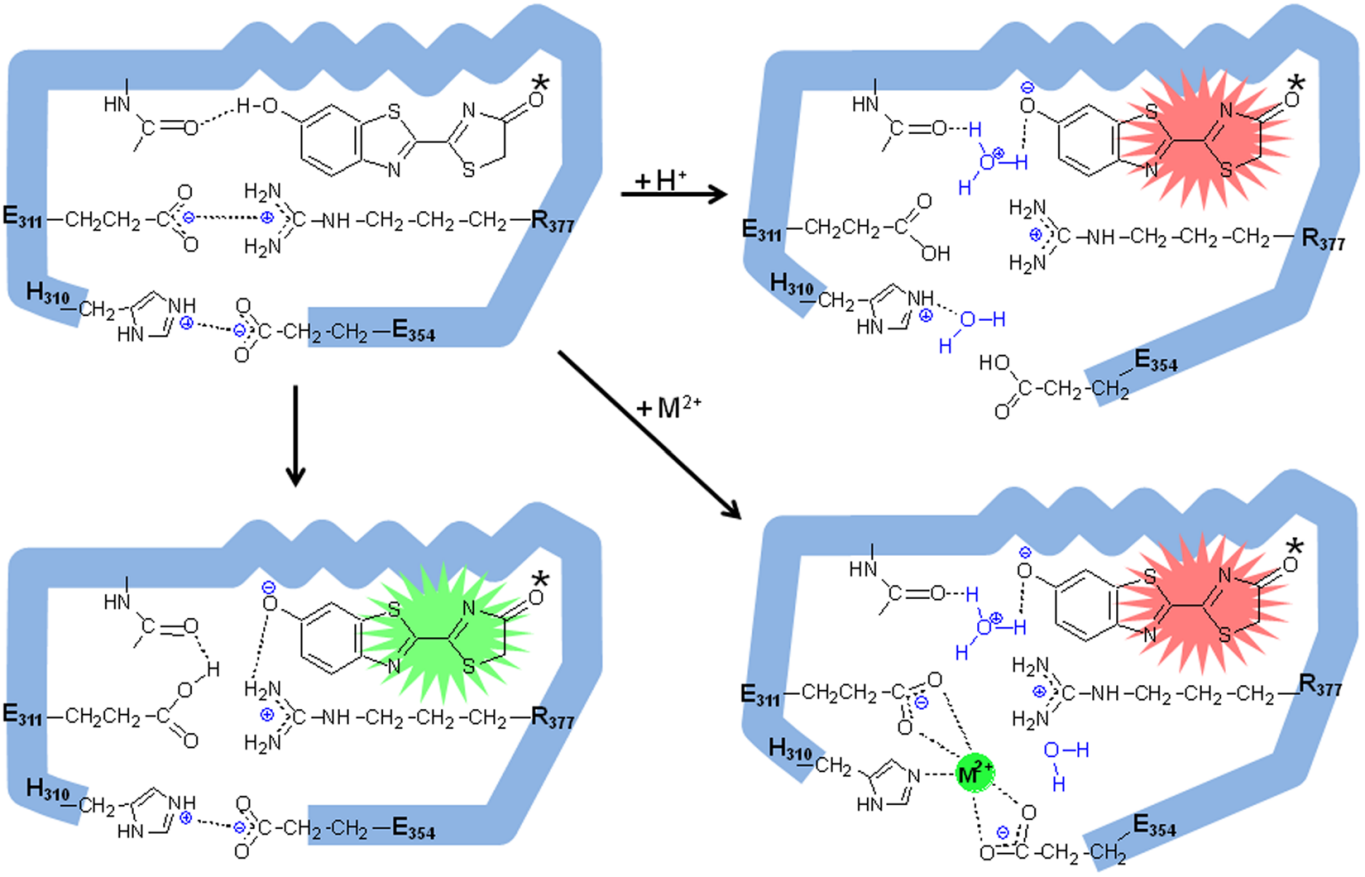
Proposed mechanism of bioluminescence color modulation by pH and divalent heavy metals in firefly luciferases. The figure is just representative, it does not necessarily show the true position of the residues.

The divalent metal ions such as Zn^2+^, Cu^2+^, Hg^2+^, Pb^2+^, Cd^2+^ clearly fit in this amphyphilic cavity, coordinating especially with E311 and E354 carboxylates, which are located close to each other (3.5 Å), and also with H310 imidazolium in most firefly luciferases (Fig. 4). The imidazole and carboxylate side chains of residues at positions 310 and 354 are responsible for modulating the sensitivity of *Macrolampis* and *Cratomorphus* firefly luciferases to metal ions such as nickel and zinc^8^. The interaction of the divalent metal ions with these glutamates in most firefly luciferases disrupt the salt bridges between E311/R337 and H310/E354, unshielding the positive charges of H310 and especially of R337, polarizing the environment (Fig. 7). Such excess of positive charges increases the polarity of the oxyluciferin phenolate binding pocket by creating a hydration shell around R337, resulting in red light emission. Noteworthy, the mutation H310R in *Macrolampis* luciferase which naturally lacks E354, partially simulates the effect of the metal, by inserting an additional positive charge in the neighborhood, and thus broadening and red-shifting the spectra.

Despite their importance for metal sensitivity, neither H310 nor E354 can be considered the only proton and metal binding sites, since H310 and E354 are not strictly conserved in other firefly luciferases which are also pH-sensitive, and their mutations by non-acid-base and residues do not abolish either pH-and metal-sensitivity. The residue H310 is substituted by threonine or valine in some firefly and other beetle luciferases. In *Macrolampis* firefly luciferase the lack of E354 negative charge upon the natural substitution E354N is responsible for the observed weaker metal sensitivity and for the naturally increased proportion of red light observed in its bioluminescence spectrum in relation to the close *C. distinctus* and *P. pyralis* firefly luciferases. Therefore, other groups could be important for metal binding, including the main chain amide bond of C/S/T314 which has been already shown to be located near oxyluciferin phenolate and to be important for bioluminescence color modulation^44^.

The internal salt bridge between E311 and R337 was previously shown to play a critical role in bioluminescence color, by keeping a closed hydrophobic conformation favorable for green light emission^55^. Although we already suggested that these residues could play additional specific functions besides the salt bridge, the effective contributions of the salt bridge in keeping a closed conformation, or specific interactions of E311 and R337 in bioluminescence color determination, remained to be elucidated. Here we showed that the exchange of these residues in the double mutant E311R/R337E, with the aim to keep the salt bridge and a closed conformation, resulted in a red emitting mutant, indicating that green light emission does not depends exclusively on a closed active site conformation, but also on more specific effects of E311 and R337.

The results shown in this manuscript bring compelling evidences that E311 carboxylate is the main base responsible for green light emission and pH-sensitivity, and the third metal binding site in firefly luciferases. The acid-base properties of E311 carboxylate, the residual pH-sensitivity upon its substitution by the conservative aspartate (E311D), the complete abolishment of pH-sensitivity upon substitution by other residues that lack negative charge (E311A/Q), provide compelling evidences that E311 carboxylate, most likely through a water molecule, acts as a main base in bioluminescence color determination. Although, the estimated pKa value of E311 carboxylate in this environment (~4.5) is apparently lower than the expected value for a critical base mediating the pH effect between 6.0–8.0, the close proximity to oxyluciferin phenol which acts as a strong acid upon chemiexcitation, may affect the pKa of E311 increasing the sensitivity of this group to external pH-changes.

Furthermore, the complete abolishment of bioluminescence activity upon charge reversal in the mutant E311R, and in the negative charge lacking mutants E311A and E311Q at pH 6.0, indicate the unforeseen possibility of a more integral role of E311 carboxylate in bioluminescence catalysis. All beetle luciferases have E311, and in most of them the negative charge of E311 carboxylate is counterbalanced by the positive charge of guanidinium group of residue R337 (only *Phrixotrix* luciferases do not). According to CTIL (charger transfer induced luminescence) mechanism, during the chemiexcitation step, the phenolic hydroxyl group of the dioxetanone intermediate must be necessarily deprotonated to give phenolate, in order to induce intramolecular charge transfer, promoting its decomposition into carbon dioxide and the excited singlet oxyluciferin which decays with emission of light^8^. The carboxylate of E311, assisted by a water molecule, may play the essential role of catalytic base for proton abstraction of the phenolic hydroxyl group of the dioxetanone intermediate, generating excited oxyluciferin phenolate during the chemiexcitation step. Because excited oxyluciferin phenol is a much stronger acid (pKa < 1.0) than E311 carboxylate (pKa ~ 4.5), it is likely that E311 carboxylate will work as a base retaining the proton. Under such circumstances, E311 carboxylate will be protonated and R337 guanidinium ion will in turn counter stabilize the negative charge of oxyluciferin phenolate. In contrast, in the electropositive microenvironment of the mutants E311R, or E311A/Q at pH 6.0, the phenol proton abstraction necessary for the generation of the critical phenolate ion during the dioxetanone intermediate activation could be halted, severely impacting bioluminescence activity.

At alkaline pH, the electrostatic couple E311/R337, reinforced by the external H310/E354 salt bridge, may work as a gate which keeps a closed conformation, as well as an electrostatic cushion that keeps the oxyluciferin ejected proton near its phenolate into a high energy state (Fig. 7). Acidic pH, the presence of heavy metals or higher temperatures weaken such electrostatic gates, decreasing the force to retain the oxyluciferin excited state released proton near its phenolate group. The few surrounding organized active site water molecules (Fig. 7) may escape as hydronium ions and be exchanged with water, polarizing and increasing the mobility of the microenvironment. Altogether these factors will decrease the energy of excited state resulting in red light emission. Similarly, in the absence of the external salt bridge between H310 and E354 in some beetle luciferase and mutants, the more internal salt bridge between E311 and R337 could be also weakened and the environment relaxed, increasing the proportion of red light. The lack of these salt bridges is observed in *Macrolampis* firefly luciferase which has E354 substituted by asparagine and produces a broader bioluminescence spectrum, and in a more extreme case, *Phrixotrix hirtus* railroadworm red emitting luciferase, which also displays asparagine at the corresponding position 351 and, and additionally lacks arginine at position 334 (L334 corresponding to R337 of *P. pyralis* luciferase), missing both salt bridges corresponding to E311/337 and H310/E354.

## Material and Methods

### Plasmids and beetle luciferases cDNAs

The cDNAs for *Macrolampis* sp2 luciferase was originally inserted in pPro vector (Invitrogen) and then was subcloned into *NdeI* site of pCold vector (Takara, Japan), the cDNA of *Cratomorphus distincus* luciferase in pBluescript vector^32^, the cDNA of *Amydetes vivianii* luciferase was cloned in pSport vector (Invitrogen)^35^.

### Site-directed mutagenesis

The mutants Mac H310A, H310C, N354E, N354C, N354H, E311A, E311Q, E311D were previously constructed^8^ by site-directed mutagenesis using an Agilent site-mutagenesis kit (Catalog 200518). The following primers were designed for generating new mutations: (E311R) CT AAT T TG CAC AGA ATT GCT TCT GG; (R337E) CCA GGT ATA GAA CAA GGA TAT GGG C. To prepare the double mutant Mac-E311R/R337E we performed mutagenesis using the mutant E311R and the primers Mac-R337E. The plasmids containing the luciferase cDNAs were amplified using *Pfu* turbo polymerase and 2 complementary primers containing the desired mutation, using a thermal cycler (1 cycle 95 °C; 25 cycles 95 °C, 30 s; 55 °C, 1 min and 68 °C 7 min). After amplification, mutated plasmids containing staggered nicks were generated. The products were treated with *Dpn* I in order to digest non-mutated parental plasmids, and used directly to transform *E. coli* XL1-Blue cells.

### Luciferase expression and purification

For luciferase expression, transformed *E. coli* BL21-DE3 cells were grown in 500–1000 mL of LB medium at 37 °C up to OD_600_ = 0.4, and then induced at 18 °C with 0.4 mM IPTG during 18 h. Cells were harvested by centrifugation at 2,500 g for 15 min and resuspended in extraction buffer consisting of 0.10 M sodium phosphate buffer, 1 mM EDTA, 1 mM DTT and 1% Triton X-100, 10% glycerol and protease inhibitor cocktail (Roche), pH 8.0, lysed by ultrasonication and centrifuged at 15,000 g for 15 min at 4 °C. The N-terminal histidine-tagged *Macrolampis sp2* luciferase was further purified by agarose-Nickel affinity chromatography followed by dialysis and anion-exchange chromatography, according to established procedures^54^. The concentrations of purified luciferases were between 0.5 and 1 mg/mL, and the estimated purity, according to SDS-PAGE gels were about 90%.

### Measurement of luciferase activity

Luciferase bioluminescence intensities were measured using a AB2200 (ATTO; Tokyo, Japan) luminometer. The assays were performed by mixing 5 μL of 40 mM ATP/80 mM MgSO_4_ with a solution consisting of 5 μL of luciferase and 85 μl of 0.5 mM luciferin in 0.10 M Tris-HCl pH 8.0 at 22 °C. All assays were measured in triplicate.

### Bioluminescence spectra

Bioluminescence spectra reported here were recorded in ATTO Lumispectra spectroluminometer (Tokyo, Japan) with cooled CCD camera whereas the previously reported spectra used the same equipment or a Hitachi F4500 spectrofluorometer. The bioluminescence spectra reported here using the CCD-based spectroluminometer are red-shifted in relation to previously reported values measured with a spectrofluorometer. Such differences are likely to be caused by distortions associated to the longer scanning time and photosensitivity correction in the older spectrofluorometer in relation to the new equipment. For the *in vitro* bioluminescence recorded using the spectroluminometer, 5.0 μL of luciferases were mixed with 90 μL of 0.10 M Tris-HCl pH 8.0, 5 μL of 10 mM D-luciferin or 6′-aminoluciferin, and 5 μL of 40 mM ATP/80 mM MgSO_4_. The pH effect on bioluminescence spectra was analyzed in 0.10 M Phosphate buffer pH 8.0 and 6.0, and 0.10 M Tris-HCl pH 8.0. The 6′-aminoluciferin analog was synthetized by T. Hirano.

### Homology Modelling

Homology-based models of *Phrixotrix hirtus*, *Pyrearinus termitilluminans, Amydetes vivianii* and *Macrolampis sp2*. luciferases were constructed using as template the three-dimensional structure of *Luciola cruciata* luciferase in the presence of DLSA (PDB code 2D1S) and of oxyluciferin and AMP (PDB code – 2D1R) as previously described^55^, Modeller v9.9 was used to align the sequences (using align2d function) and to construct 200 three dimensional models of each sequence Visualization and analyses of the best model of each luciferase were performed using PyMol version 1.4.1^60^.

### pKa estimation and system neutralization

Initially, side-chain conformations for pH-sensor residues were optimized using SCWALL method of Optimize function in YASARA^61^. To achieve theoretical pKa values for each residues of studied luciferase we used the routine “Experiment Neutralization” of software YASARA^62^. Initially, the hydrogen bonding network was optimized^61^. The method to predict pKa is based on a empirical function that uses hydrogen bonds, Coulomb potential and surface accessible area parameters weighted by experimental data. Electrostatic terms are calculated using Ewald summation^63,64^ using force field AMBER03. pKa values for Asp, Glu, His and Lys residues were predicted on the pH 6.0 and 8.0. During model construction, protonation states were assigned using these rule: Protonate Asp and Glu if the predicted pKa is higher than the pH; Protonate His if the predicted pKa is higher than the pH and His is not a hydrogen bond acceptor or deprotonate otherwise. Cys is deprotonated if the selected pH is higher than 8.7. Deprotonate Lys if the predicted pKa is lower than the pH. After that, simulation box was filled with water molecules, and ions are placed at the positions to neutralize highest and lowest electrostatic potential residues. A short MD simulation was ran for the solvent, and water molecules are subsequently deleted until the water density has reached 1.0 g/ml.

### Potential Energy calculation

To calculate the potential energy of the selected residues using parameters from force field Amber03 and cutoff distance of 8.0 A. Energies were calculated for wild-type luciferases of *Amydetes*, *Cratomorphus*, *Macrolampis* and *Phrixotrix*. Statistical software R^65,66^ was used to create a script that processed energy data for each pH-sensor residue. To check the combination of residues that better explained the spectrum shift, the energies of four residues were combined in pairs and trios, creating eleven microenvironment putative potentials. Using linear model that relate emission wavelength of a given luciferase against each microenvironment potential, it was defined the best combination using the explained variance statistic (R2). The results are plotted using ggplot2 library^67^.

## Concluding Remarks

The proton and metal binding site responsible for pH- and metal-sensitive bioluminescence color change in firefly luciferases involves mainly the side-chains of H310, E311 and E354 near the oxyluciferin phenolate binding site. These residues are involved in two critical salt bridges (E311/R337 and H310/E354) which keep oxyluciferin phenolate binding site electrostatically closed, promoting green light emission. The carboxylate of E311 is the main base responsible for green light emission and pH-sensitivity, playing also a critical role as a water mediated catalytic for bioluminescence activity base, by assisting proton abstraction of the dioxetanone intermediate phenol group during the chemiexcitation step. Under physiological and alkaline pH conditions, the stable salt bridge between the residues E311 and R337, aided by the more external salt bridge between E354 and H310 (in many firefly luciferases), keep a closed hydrophobic active site environment retaining the excited oxyluciferin released proton near its phenolate group into a high energy state, promoting green light emission. At lower pH or upon metal binding to H310, E311 and E354, the salt bridge between E311 carboxylate and R337 guanidinium is disrupted, reducing the ability of E311 to retain the excited oxyluciferin released proton near its phenolate group, polarizing the environment and thereof resulting in red light emission.

## Electronic supplementary material

Supplementary Fig.8

Table 2
