# Supplementary material for: The proton and metal binding sites responsible for the pH-dependent green-red bioluminescence color tuning in firefly luciferases
Source: Sci Rep. 2018 Dec 4;8:17594. doi: 10.1038/s41598-018-33252-x (PMC6279810; doi:10.1038/s41598-018-33252-x)
Supplement: Supplementary file 1 — Supplementary Fig.8 [file 41598_2018_33252_MOESM1_ESM.docx]

**The proton and metal binding sites responsible for the pH-dependent green-red bioluminescence color tuning in firefly luciferases**

Vadim R. Viviani^*†^, Gabriele V. M. Gabriel^⊥^, Vanessa R. Bevilaqua^⊥^, A. Simões^†^, T. Hirano^¥^, P. S. Lopes-de-Oliveira^#^

**Figure 8**. Bioluminescence spectrum of *Macrolampis* sp2 luciferase mutant E311R/R337E.
