# Supplementary material for: The proton and metal binding sites responsible for the pH-dependent green-red bioluminescence color tuning in firefly luciferases
Source: Sci Rep. 2018 Dec 4;8:17594. doi: 10.1038/s41598-018-33252-x (PMC6279810; doi:10.1038/s41598-018-33252-x)
Supplement: Supplementary file 2 — Table 2 [file 41598_2018_33252_MOESM2_ESM.docx]

**The proton and metal binding sites responsible for the pH-dependent green-red bioluminescence color tuning in firefly luciferases**

Vadim R. Viviani^*†^, Gabriele V. M. Gabriel^⊥^, Vanessa R. Bevilaqua^⊥^, A. Simões^†^, T. Hirano^¥^, P. S. Lopes-de-Oliveira^#^

**Table 2.** Effect of mutations on the pKa values and potential of *Macrolampis* and *Cratomorphus* firefly luciferases. (Supplemental materials)

| **Luciferase/**  **Mutant** | **pKa**  **H310** | **pKa**  **E311** | **pKa**  **R337** | **pKa**  **E/N354** | **Overall charge**  **pH 8.0** | **Overall charge**  **pH 6.0** | **λ_max_/**  **[Band.] (nm) **** |
| --- | --- | --- | --- | --- | --- | --- | --- |
| *Macrolampis* WT | 7.85 | 4.53 | 13 |  | 0 | 1 | 573 [99] |
| H310A | - | 3.54 | 13 | - | 0 | 0 | 578 [99] |
| H310C | 8.7 | 3.54 | 13 | - | 0 | 0 | 573 |
| H310R | 13 | 3.3 | 13 | - | 1 | 1 | 573 [105] |
| E311A | 4.93 | - | 13 | - | 1 | 2 | 621 |
| E311Q | 4.86 | - | 13 | - | 1 | 2 | 623 [67] |
| E311D | 5.38 | 2.62 | 12 | - | 0 | 1 | 600 [63] |
| R337K | 5.51 | 3.92 | 10.79 | - | 0 | 1 | 606 [63] |
| R337E | 8.57 | 4.4 | 4.65 | - | -2 | -1 | 597 [93] |
| N354E | 6.84 | 4.11 | 13 | 4.55 | -1 | 0 | 558 [83] |
| N354C | 7.13 | 3.89 | 13 | 8.7 | 0 | 1 | 564 |
| N354H | 7.11 | 3.68 | 13 | 7.12 | 0 | 2 | 568 |
| *Cratomorphus* | 8.9 | 5.0 | 13 | 4.65 | -1 | 0 | 548 [71] |
| E354N | 8.14 | 4.76 | 13 |  | 0 | 1 | 556 [86] |
